# Multiple lumbar transverse process stress fractures as a cause of chronic low back ache in a young fast bowler - a case report

**DOI:** 10.1186/1758-2555-3-8

**Published:** 2011-04-10

**Authors:** Kamal Bali, Vishal Kumar, Vibhu Krishnan, Dharm Meena, Saurabh Rawall

**Affiliations:** 1 Deptt of Orthopaedics, PGIMER, Chandigarh, India; 2 Deptt of Orthopaedics, AIIMS, New Delhi, India; 3 Postgraduate Institute of Medical Education and Research, Sector 12, Chandigarh- 160 012, India

## Abstract

A rare case of multilevel transverse process stress fractures as a cause of low back ache in a professional cricket player has been presented. The report discusses the possible mechanism of such an injury in a cricket player and also highlights the preventive and therapeutic aspects of management in such patients. The report also stresses upon the need for early identification of such sports related injuries to prevent long term morbidity in the athletes.

## Introduction

Cricket, though long heralded as a "gentleman's game" [1], has evolved into shorter and more competitive versions involving greater aggression, more stressful training programmes and heavier workload on the athletes on par with any other professional sports. This has expectedly ensued in an increase in the number of cricketing injuries lately: broadly classified into collision injuries (direct contact) and overuse injuries [1]. The modern protective gadgets, lately available universally to cricketers, have greatly reduced the collision injuries, notwithstanding a relative, steady rise in the incidence of the overuse injuries. The spine, described as central pillar of the body [2,3], bears a major brunt of these athletic trauma. The present article deals with a fast bowler (cricketer) who had presented to us with chronic low-back ache following displaced stress fractures of multiple transverse processes of lumbar vertebrae. We discuss uniqueness in the mechanism of such injuries and expatiate on the preventive and treatment aspects of management.

## Case report

A twenty-six years old young cricketer (an amateur right handed fast bowler who was playing at the state level with an average of 7 to 8 matches at the state and club levels every month and 2-3 hours of training on an average daily) presented to the out-patient department of our hospital in August 2009 with complaint of pain in lower back for three years. He had received long, albeit unsuccessful treatments (predominantly with NSAIDs and physiotherapy). There was no history of sensory, motor or autonomic deficits. On further inquiry, the patient came up with a history of slip while bowling around three years back. However, the injury sustained was insignificant according to him. There was no history of loss of consciousness, ear or nasal bleed, seizures, abdominal pain, backache at the time of initial trauma, though minor skin abrasions were allegedly present. Also, as per the history, there was no relation of the onset of pain with the injury sustained as the injury occurred around 5 months before the pain actually started.

Initially, the pain was gradual in onset, typically aggravated on activities and relieved with rest and medications. At the time of presentation, the pain was so severe that it not only hampered his athletic career, but also had significantly affected his activities of daily living. The pain was localised to the lower back and the over the left flank.

On examination, the vital parameters and systemic examinations including respiratory, central and peripheral nervous, cardiovascular systems and abdomen were normal. Motor power, sensory examination and reflexes (deep tendon and superficial) of all the four limbs were found to be normal. There was a mild scoliotic deformity with convexity to the left side and deep tenderness in left paraspinal region. Repeated abdomen ultrasounds were performed and reported to be normal.

**Figure 1:**
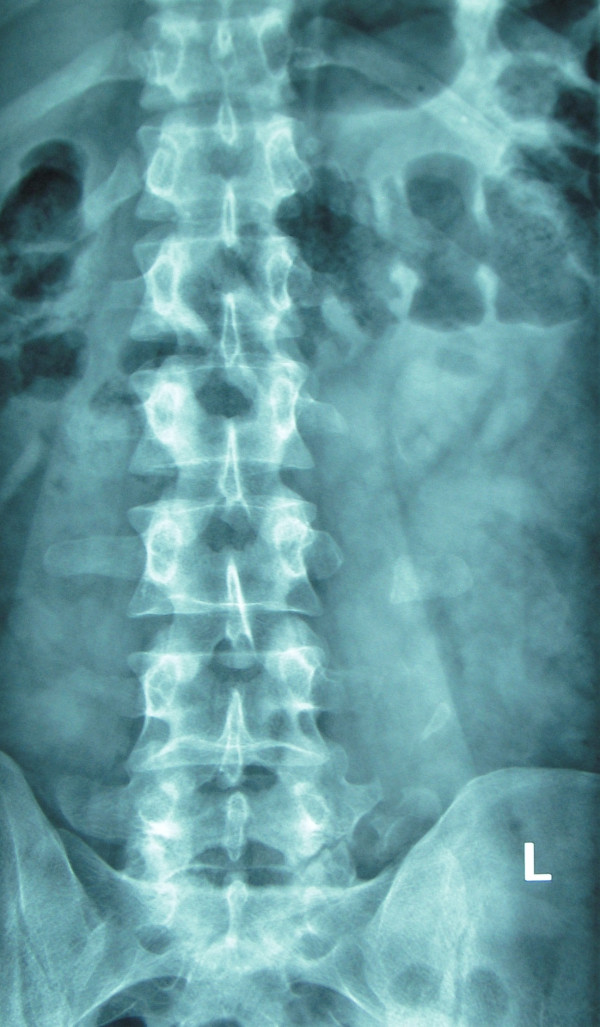
**AP radiograph of the patient showing multiple level transverse process stress fractures**.

**Figure 2:**
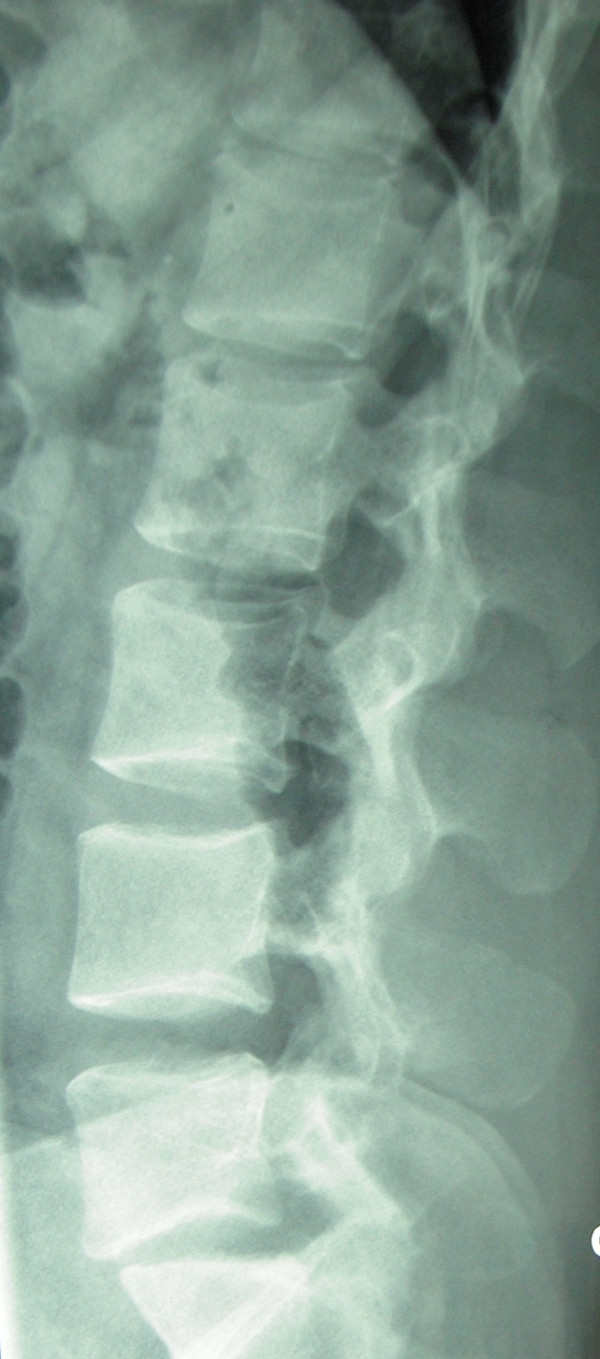
**Lateral radiograph of the same patient**.

**Figure 3:**
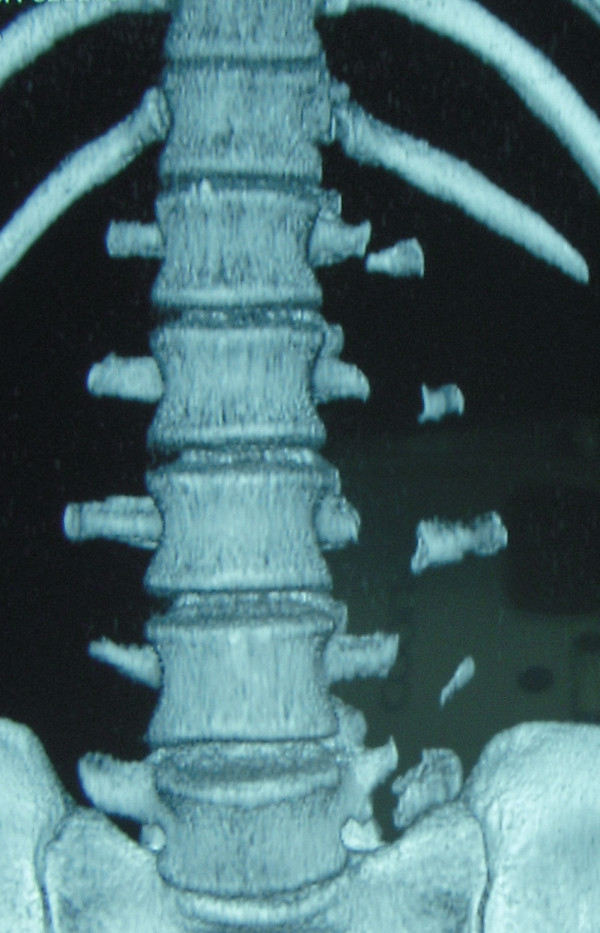
**CT scan with 3-D reconstruction image of the same patient; anterior aspect**.

**Figure 4:**
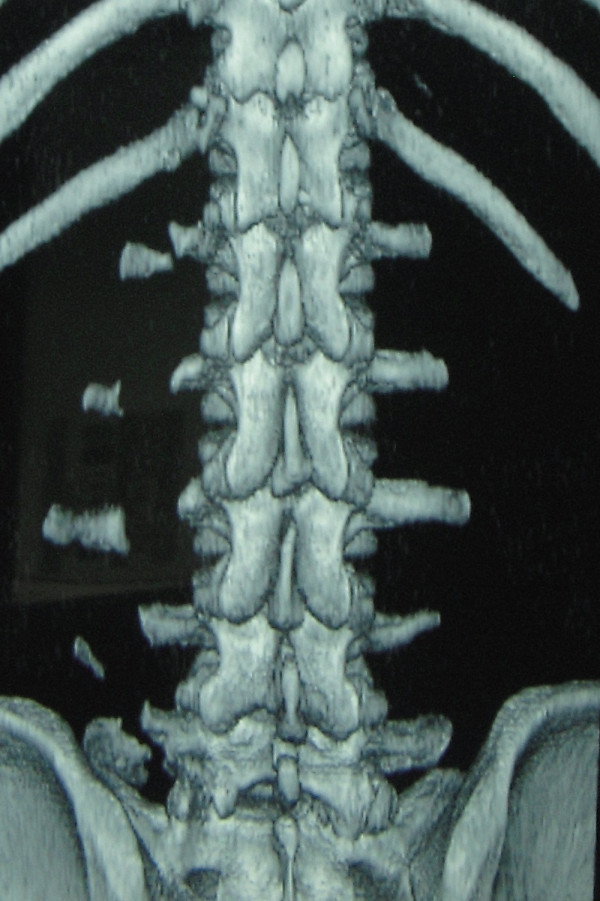
**CT scan with 3-D reconstruction image of the same patient; posterior aspect**.

X rays of abdomen and lumbosacral spine were carried out that revealed fracture non-union of L1 to L5 vertebral transverse processes on left side (Figure 1 and Figure 2). Non-contrast CT scan confirmed the diagnosis of displaced fracture non-union from L1 to L5 transverse processes on the left side (Figure 3 and Figure 4). As the fracture pattern looked terminal, bony union was going to be highly unlikely and the patient was counselled about the same. The goal of the treatment was rehabilitation of the patient and gradual return to active sports once the acute episode of pain settled. Ice application initiated to start with. The patient was also advised strict restraint from any sort of sporting activity for 4-6 weeks and ultrasonic massage therapy carried out initially. Specific spine strengthening exercise protocol as elaborated later in the article was initiated progressively and the player was gradually allowed to play with a spinal brace for a period of 5 months.

The patient has gradually managed to recuperate and after the rehabilitation regimen (extending over 9-10 months), is currently able to participate in sporting activities, though with occasional episodes of backache. Back radiographs at the last follow up, however, continue to show the evidence of non union of the left L1 to L5 vertebral transverse fractures.

## Discussion

Spine injuries constitute 7% of cricket-related trauma [1], with fast bowlers being most prone to these injuries. The most common bony abnormalities following overuse injury in the spine in fast bowlers are: spondylolysis, spondylolisthesis and pedicle sclerosis [4], with ligament sprains, muscular strains and disc degenerative disorders being the other notable causes of low back ache. These spine fractures also occur more commonly in the younger athletes with relatively immature spine [1]. The present article elaborates on an unusual sports-related fracture of the spine involving the avulsion of multiple transverse processes of lumbar vertebrae.

Most athletes who sustain a low back injury do so while lifting heavy weights or while performing unexpected, sudden coupled motions [5] (eg, lateral bending and flexion, lateral bending and axial rotation). Risk factors for the development of low back pain or injury in athletes [6,7] include:

1. Muscular imbalances or weaknesses of the abdominal and posterior spinal muscles.

2. Deficits in the afferent or efferent pathways or proprioceptors.

3. Preexisting structural deformities, such as scoliosis, spondylolysis, or spinal fusions.

The low back pain in the athletes (especially following stress fractures of vertebrae) is described as crescendo-type pain [8] that typically occurs towards the end of the bowling spell initially, progressing gradually, occuring at an earlier time as days progress until it occurs right at the middle of the spell one fine day.

Fast bowling action involves repetitive movements including twisting, extension and rotation of the trunk within a short period [1]. The bowling action includes three stages that occur sequentially: run-up to back foot impact, delivery stride and release. Of these, delivery stride is the most important phase, during which unnatural postures and trunk misalignments may severely augment the stresses on the spine contributing to injuries (although spine injuries are known to occur in the other phases of bowling too). This phase is further segregated into three different segments: stride length, stride alignment and shoulder alignment, of which the latter two are the significant predisposing factors to lumbar spine stress injuries [1].

A bowler [1] may adopt one of the following techniques of delivering the ball: side-on, front-on or mixed action. The side-on action involves a shoulder alignment of 190 degrees or less and a back-foot angle of 280 degrees or less and places the least amount of stress on the lower back (as it invoves the least extension and lateral flexion movements of the trunk). The front-on technique involves a shoulder alignment greater than 190 degrees and back-foot angle greater than 280 degrees. The mixed action is a combination of these techniques involving excessive twisting of the trunk, thus leading onto the adoption of hyperextended and laterally flexed position of spine. An athlete with this type of bowling action is, especially, prone to significant overuse injuries of the spine [1]. Even our patient had a mixed action of bowling that could have predisposed him to his spine injury.

Fracture of the transverse process of vertebra was long believed to be a minor, stable fracture with little need for intervention. However, studies have suggested that these injuries typically follow high energy trauma and heavy impact, thereby are commonly accompanied by significant associated injuries (eg. intra-abdominal injuries) [9]. This fracture, however, follows a much different mechanism in athletic stress fractures, involving much less significant forces [10].

In cricket, these transverse process fractures may either result from collision injuries; for example following a direct impact from cricket ball, stress (overuse) fractures following excessive, repetitive contractions of major trunk muscles (especially in fast bowlers) or acute spasmic contractions of the lower trunk muscles, with ensuing transverse process avulsion. The two major muscles believed to be acting on the lumbar transverse processes are quadratus lumborum, which originates from the 12th rib and tips of the transverse processes L1-L5, and psoas which originates from the anterior surfaces of the lumbar transverse processes. A recent cadaveric assessment of the attachments of the lumbar transverse processes revealed that the attachment of transversus abdominis muscle through the middle layer of lumbar fascia may play a major role in causing these avulsion fractures [11,12]. Although our patient gave a history of slip, the injury was not believed to be significant enough and it was the repeated small stresses on the transverse processes due to the nature of the sports in the athlete (fast bowling) that ultimately lead to the presenting picture in the patient.

Our patient also had a non-structural scoliotic deformity, convex to the same side as the transverse process fracture. This may be attributed to the ineffective action of the ipsilateral quadratus lumborum muscle with concomitant unopposed contraction of the contralateral quadratus lumborum [13]. The stress fractures of the spine also predispose to hamstring spasm, thereby, aggravating the discomfort of the athlete.

The diagnosis of this injury was quite simple and straight-forward in our case. However, as reported by a few authors [12,14], this might not always be the case and the injury is liable to be missed if a high degree of suspicion is not kept. The plain radiographs provide the most useful, initial investigation that may be supplemented by additional informations provided by other confirmatory investigations like technicium 99m bone scintigraphy and computerised tomography. However it is the magnetic resonance imaging (MRI) that is turning out to be the one of the most valuable tools these days to detect stress fractures especially at an early stage.

In most cases, complete rest from sports is the treatment of choice [1,2,4,5]. The time required for the fracture to heal is usually 6 weeks. Even the cases of nonunion also settle with time and become asymptomatic. During this time progressive rehabilitation program is initiated, that involves strengthening of the structures supporting the lumbar spine like the transversus abdominis, multifidus, spinal erectors and hip abductors. Exercises for improving the core stability of the trunk and flexibility of the trunk and lower extremity are also undertaken. A brace to support the back while bowling may also be advocated over the initial 3-4 months.

The best way [1,2,4,5,15] to ensure pain-free careers in the athletes is by taking the fundamental and necessary steps in avoiding the spine injuries in the athletes: the most important of which is to ensure a proper rotation policy in the team and ensure tolerable workloads especially in the younger athletes. Proper specialised fitness programmes aimed at strengthening the mid-section and lower extremity musculature and ameliorating flexibility need to be ensured. Mixed actions of bowling that are colossally fraught with spine injuries need to be modified at an early part of an athlete's career.

## Conclusion

A high index of suspicion is cardinal to identify such injuries at the right time. These injuries, though relatively minor, add significantly to the morbidity of the patients. The right diagnosis at the right time salves the athlete's problems and enhances his productivity.

## Consent

Written informed consent was obtained from the patient for publication of this case report and any accompanying images. A copy of the written consent is available for review by the Editor-in-Chief of this journal.

## Conflict of interests

The authors declare that they have no competing interests.

## Authors' contributions

Dr KB and Dr SR reviewed the literature and wrote the paper. Dr VK_1 _and Dr VK_2 _maintained all the records of the patient and followed him. Dr KB and Dr DM were incharge of the rehabilitation protocol of the patient. All the authors read and approved the final manuscript.
